# Supplementary material for: Time-series transcriptome analysis identified differentially expressed genes in broiler chicken infected with mixed Eimeria species
Source: Front Genet. 2022 Aug 8;13:886781. doi: 10.3389/fgene.2022.886781 (PMC9393255; doi:10.3389/fgene.2022.886781)
Supplement: Supplementary file 2 [file DataSheet1.ZIP › 7dpi_KEGG.Gsea.1625115961950/KEGG_ARACHIDONIC_ACID_METABOLISM.html]

Details for gene set KEGG\_ARACHIDONIC\_ACID\_METABOLISM[GSEA]

|  || Dataset | TMM\_7dpi\_gct\_format\_7dpi\_gct\_format.Class\_4dpi.cls #PC\_versus\_NC.Class\_4dpi.cls #PC\_versus\_NC\_repos |
| Phenotype | Class\_4dpi.cls#PC\_versus\_NC\_repos |
| Upregulated in class | 1 |
| GeneSet | KEGG\_ARACHIDONIC\_ACID\_METABOLISM |
| Enrichment Score (ES) | 0.3321391 |
| Normalized Enrichment Score (NES) | 1.229829 |
| Nominal p-value | 0.1576414 |
| FDR q-value | 0.41185248 |
| FWER p-Value | 0.9892 |
Table: GSEA Results Summary

  

Fig 1: Enrichment plot: KEGG\_ARACHIDONIC\_ACID\_METABOLISM      
 Profile of the Running ES Score & Positions of GeneSet Members on the Rank Ordered List

  

| SYMBOL | TITLE | RANK IN GENE LIST | RANK METRIC SCORE | RUNNING ES | CORE ENRICHMENT || 1 | GPX1 | na | 190 | 1.058 | 0.0555 | Yes |
| 2 | HPGDS | na | 209 | 1.032 | 0.1236 | Yes |
| 3 | PLA2G2E | na | 298 | 0.942 | 0.1797 | Yes |
| 4 | PLA2G5 | na | 299 | 0.942 | 0.2432 | Yes |
| 5 | PTGDS | na | 416 | 0.806 | 0.2878 | Yes |
| 6 | CYP2C18 | na | 638 | 0.669 | 0.3145 | Yes |
| 7 | GPX4 | na | 881 | 0.561 | 0.3321 | Yes |
| 8 | CYP4A22 | na | 1398 | 0.407 | 0.3165 | No |
| 9 | PTGES2 | na | 1658 | 0.342 | 0.3180 | No |
| 10 | GPX7 | na | 3120 | 0.112 | 0.2036 | No |
| 11 | GPX3 | na | 3522 | 0.061 | 0.1743 | No |
| 12 | PLA2G6 | na | 4448 | -0.045 | 0.1001 | No |
| 13 | EPHX2 | na | 4581 | -0.059 | 0.0930 | No |
| 14 | CBR3 | na | 6059 | -0.209 | -0.0161 | No |
| 15 | PTGES | na | 6833 | -0.290 | -0.0611 | No |
| 16 | GGT7 | na | 6952 | -0.302 | -0.0505 | No |
| 17 | PTGS2 | na | 6986 | -0.306 | -0.0327 | No |
| 18 | PLA2G4A | na | 7107 | -0.320 | -0.0211 | No |
| 19 | PTGS1 | na | 7476 | -0.359 | -0.0276 | No |
| 20 | PLA2G4B | na | 7552 | -0.367 | -0.0092 | No |
| 21 | CYP2U1 | na | 9307 | -0.600 | -0.1150 | No |
| 22 | PLA2G12B | na | 9771 | -0.669 | -0.1086 | No |
| 23 | GGT5 | na | 10882 | -0.903 | -0.1403 | No |
| 24 | LTA4H | na | 11367 | -1.066 | -0.1088 | No |
| 25 | TBXAS1 | na | 11450 | -1.098 | -0.0417 | No |
| 26 | ALOX5 | na | 11781 | -1.310 | 0.0191 | No |
Table: GSEA details [plain text format]

  

Fig 2: KEGG\_ARACHIDONIC\_ACID\_METABOLISM      
 Blue-Pink O' Gram in the Space of the Analyzed GeneSet

  

Fig 3: KEGG\_ARACHIDONIC\_ACID\_METABOLISM: Random ES distribution      
 Gene set null distribution of ES for **KEGG\_ARACHIDONIC\_ACID\_METABOLISM**
